# EGFR Gene Copy Number as a Prognostic Marker in Colorectal Cancer Patients Treated with Cetuximab or Panitumumab: A Systematic Review and Meta Analysis

**DOI:** 10.1371/journal.pone.0056205

**Published:** 2013-02-18

**Authors:** Zheng Jiang, Chunxiang Li, Fuyuan Li, Xishan Wang

**Affiliations:** 1 Department of Colorectal Cancer Surgery, The 2nd Affiliated Hospital, Harbin Medical University, Harbin, Heilongjiang, China; 2 Colorectal Cancer Institute of the Heilongjiang Academy of Medical Sciences, Harbin, Heilongjiang, China; 3 Laboratory of Medical Genetics, Harbin Medical University, Harbin, Heilongjiang, China; 4 Department of Biochemistry and Molecular Biology, Harbin Medical University, Harbin, Heilongjiang, China; Shanghai Jiao Tong University School of Medicine, China

## Abstract

**Background:**

The epidermal growth factor receptor (EGFR) gene copy number (GCN) has been previously demonstrated to correlate with the clinical outcome of colorectal cancer (CRC) treated with anti-EGFR monoclonal antibodies (mAbs), although it remains controversial. We conducted a systematic review and meta-analysis to assess EGFR GCN as a potential biomarker of survival for patients with advanced CRC receiving treatment with anti-EGFR mAbs.

**Methods:**

We systematically identified articles investigating EGFR GCN by fluorescent or chromogenic *in situ* hybridization or other detection techniques in patients with metastatic CRC treated with panitumumab or cetuximab, (last search: 10 August 2012). Eligible studies had to report on overall survival (OS), progression-free survival (PFS) or time-toprogression (TTP), stratified by EGFR GCN. Summary hazard ratios (HRs) were calculated using random-effects models.

**Results:**

Among 13 identified studies, 10 (776 patients, 302 with increased GCN), 8 (893 patients, 282 with increased GCN) and 3 (149 patients, 66 with increased GCN) were eligible for the OS, PFS and TTP meta-analyses, respectively. Increased EGFR GCN was associated with increased OS (HR = 0.62; 95% CI 0.50–0.77; *P*<0.001), PFS (HR = 0.65; 95% CI 0.47–0.89; *P* = 0.008) but not TTP (HR = 0.71; 95% CI 0.44–1.14; *P* = 0.157). It was also shown that EGFR GCN is independent of other factors such as KRAS status. Among those populations received second-line or higher treatment, increased EGFR GCN was strongly associated with improved survival (for OS, HR = 0.60; 95% CI 0.47–0.75; P<0.001; for PFS, HR = 0.59; 95% CI 0.47–0.75; P<0.001), whereas it did not influence survival in patients that received first-line therapy.

**Conclusion:**

Among the anti-EGFR-treated patients, increased EGFR GCN appears to be associated with improved survival outcomes. The effect on survival appears to be related to patients receiving the line of treatment.

## Introduction

The major prognostic determinant for patients with non-resectable metastic colorectal cancer (CRC) is the response to systemic therapy [1]. During these last years, novel strategies that target the epidermal growth factor receptor (EGFR) have been evaluated in CRC, including monoclonal antibodies (mAbs). These mAbs interfering with the extracellular domain of EGFR, were designed to be used when other treatments failed [2]. Two such mAbs, panitumumab and cetuximab, are active in metastatic colorectal cancer, but the clinical evidence shows that approximately 10% of patients achieve an objective tumor response to anti-EGFR mAbs [2]–[4]. The identification of patients who are likely to be benefited from EGFR-targeted mAbs is increasingly crucial for improving therapeutic strategies, as well as for reducing the financial burden of health care systems [5]. Therefore, the reliable prognostic markers of treatment for selected patients need to be identified.

Several clinical studies have shown that the presence of a KRAS mutation is a significant predictor of resistance to anti-EGFR mAbs [6]–[8]. However, the occurrence of KRAS mutations only accounts for approximately 30% to 40% of nonresponsive patients, suggesting that it may not be the only predictor of cetuximab response and the identification of additional genetic determinants of treatment benefits, still need to be defined. Recently, studies have demonstrated that an increased EGFR gene copy number (GCN), analyzed by the fluorescence in situ hybridization (FISH) technique, could be a promising predictor of anti-EGFR mAbs therapy in metastatic colorectal cancer, patients with low GCN are indeed unlikely to respond to anti-EGFR treatment and have less progression-free time than patients with increased GCN [9]–[12].

Moroni et al. [9] first reported an increased EGFR GCN association with a favorable response to anti-EGFR therapy among KRAS wild-type CRC patients. However, subsequent studies revealed the conclusion remained inconsistent [10], [12]. Studies on the EGFR GCN had shown different trends of the prognosis in CRC, this might be due to a relatively small size and different patient population. Therefore, it is highly necessary to perform a quantitative and systemic study with rigorous methods. Meta-analysis is a powerful means of resolving disparate results. To address the association between variations of EGFR GCN and the survival outcomes of metastatic CRC patients receiving anti-EGFR therapy, a meta-analysis was performed from all eligible studies in this study.

## Materials and Methods

### Identification and Eligibility of Relevant Studies

We performed a systematic computerized search of the MEDLINE (PubMed) database, EMbase and the Cochrane library (last search: August 10, 2012) to identify all published articles related to the identification of mutations in EGFR pertaining to CRC, using the algorithm: (epidermal growth factor receptor OR EGFR) AND (mutation OR polymorphism OR gene copy number OR GCN OR amplification OR gene status) AND (colorectal cancer OR CRC). Additional studies were identified by a hand search of references of original studies or review articles on this topic. Eligible studies included in the meta-analysis had to meet the following criteria: (a) a cohort colorectal cancer study; (b) hazard ratios (HRs) with corresponding 95% confidence intervals (CIs) comparing overall survival (OS), progression-free survival (PFS) or time-to-progression (TTP) stratified by EGFR gene copy number for patients receiving mono- or combination therapy with either cetuximab or panitumumab were reported or allowed the calculation; and (c) written in English.

### Data Extraction

Two investigators independently extracted data and reached a consensus on all of the items. The following information was extracted from each study: first author, years of publication, number of patients screened, ethnicity of study population, gender, proportion of increased GCN, the specific methods of gene copy number determination were recorded, as were the values for GCN cutoff, KRAS status, anti-EGFR mAbs, study design and also for data linking specific mutation to treatment outcome. Also, we categorized studies by line of treatment. When studies were conducted in mixed treatment settings, we operationally defined studies where at least 80% of patients had received previous chemotherapy as ‘second-line’ studies. Finally, we extracted HRs and their variance for the relevant survival outcomes comparing patients with increased and normal EGFR gene copy number receiving treatment with either cetuximab or panitumumab. The HR is the most appropriate metric for time-to-event outcomes [13], [14]. When the HR and/or its variance were not provided by the eligible studies, we used the methods developed by Parmar *et al.* [14] to calculate them. When *P* values were unavailable, the HR was approximated by the ratio of median survivals [15]. Data was extracted by the Engauge Digitizer version 4.1 (free software downloaded from http://sourceforge.net) from survival curves if it was not shown in articles directly, then we estimated the log HR and its variance using the previously reported methods [14], [16]. Two authors performed data extraction independently and discrepancies were resolved by consensus including a third author.

### Statistical Analysis

We used the HR and corresponding CI extracted from each study to assess between-study heterogeneity using the Q statistics [17] and inconsistency using the *I* ^2^ index [18] (*I* ^2^<25% no heterogeneity; *I* ^2^ = 25–50% moderate heterogeneity; *I* ^2^>50% large or extreme heterogeneity). The heterogeneity was considered statistically significant with *P*<0.10. Summary HRs with their 95% CI were calculated using an inverse variance method. We fitted a random-effects model since between-study heterogeneity was anticipated [19]. Publication bias was investigated by funnel plot, and an asymmetric plot suggested possible publication bias. The funnel plot asymmetry was assessed by Egger’s linear regression test [20]. The t test was performed to determine the significance of the asymmetry, and a *P* value of <0.05 was considered a significant publication bias.

Subgroup analyses were performed to evaluate the effect of ethnicity (East Asian versus white), method of EGFR GCN determination (FISH versus chromogenic *in situ* hybridization (CISH)), KRAS status (wild versus mixed), the specific EGFR mAbs used (cetuximab versus panitumumab) and line of treatment (≥80% versus <80% second-line) on the prognostic value of EGFR GCN.

Statistical analyses were conducted with Stata (version SE/10; StataCorp, College Station, TX). *P* values for all comparisons were two-tailed and the statistical significance was defined as *P*<0.05 for all tests except those for heterogeneity.

## Results

### Eligibility

**Figure 1:**
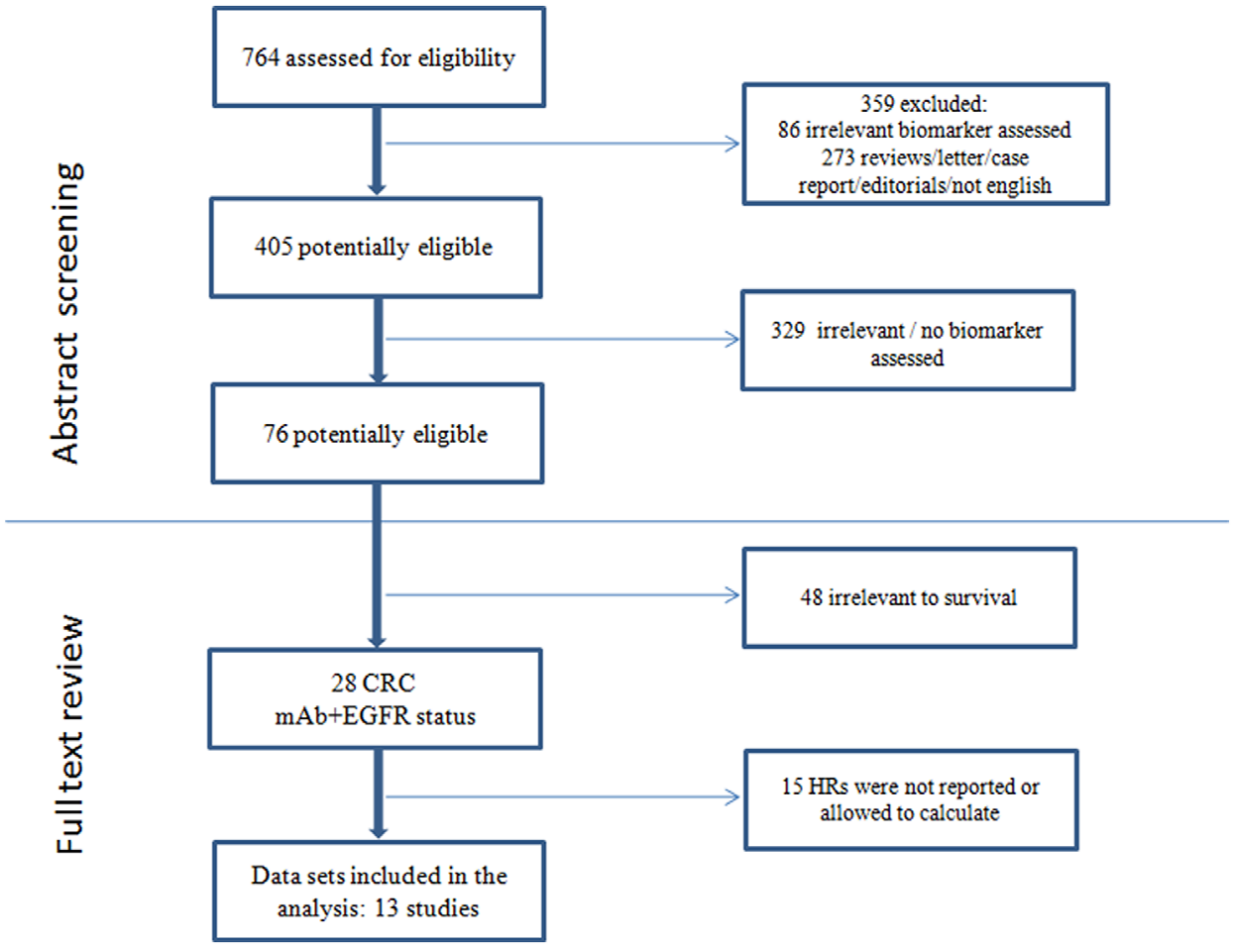
Search strategy and study eligibility flow chat.

Our initial search yielded 76 studies concerning EGFR-targeted treatment in CRC, which were assessed in full text. As indicated in the search ﬂow diagram (Figure 1), 13 studies reported at least one of the outcomes of interest and were finally included in the meta-analysis [10]–[12], [21]–[30]. The search ﬂow diagram is summarized in Figure 1 and the characteristics of eligible studies are summarized in Table 1.

**Table 1:** Characteristics of studies included in the meta-analysis.

Auther (year) reference	Patients (N)	Ethnicity	Gender(M/F)	Increased EGFR gene copy number, n (%)	Detection method	GCNcutoff	KRAS status	anti-EGFR mAbs	Line of treatment (proportion of second-line orhigher)	Study design	Outcome reported
Sartore-Bianchi (2007) [10]	58	White	33/25	19(33)	FISH	2.5	NR	panitumumab	≥80%	retrospective	OS,PFS
Cappuzzo (2008) [12]	85	White	54/31	43(51)	FISH	2.92	NR	cetuximab	≥80%	retrospective	OS,TTP
Gonçalves (2008) [21]	20	White	10/10	8(40)	FISH	2	mixed	cetuximab	≥80%	retrospective	TTP
Personeni (2008) [11]	87	White	49/38	33(38)	FISH	2.83	mixed	cetuximab	≥80%	prospective	OS,PFS
Scartozzi (2009)[22]	44	White	23/21	15(40)	FISH/CISH	2.6/2.12	wild-type	cetuximab	≥80%	retrospective	OS,TTP
Laurent-Puig (2009) [23]	96	White	NR	17(22)	FISH/CISH	2/NR	wild-type	cetuximab	≥80%	retrospective	OS,PFS
Li (2010) [24]	74*	East Asian	43/31	53(77)	FISH	2	mixed§	cetuximab	≥80%	retrospective	OS,PFS
Tol (2010) [25]	277	White	NR	41(15)	FISH	#	mixed	cetuximab	<80%	retrospective	PFS
Campanella (2010) [26]	101	White	62/39	56(56)	FISH	2	mixed	cetuximab	<80%	retrospective	PFS
Bengala (2010) [27]	146	White	96/60	29(20)	FISH	2.9	mixed	NR	<80%	retrospective	OS,PFS
Scartozzi (2011)[28]	90	White	59/31	43(48)	CISH	2.12	wild-type	cetuximab	≥80%	retrospective	OS
Lin (2011) [29]	42	East Asian	19/23	16(38)	qPCR	1.5	mixed	cetuximab	≥80%	retrospective	OS
Algars (2011) [30]	54	White	30/24	34(63)	SISH	4	mixed§	mixed	≥80%	retrospective	OS,PFS

* of 74 patients, EGFR FISH analysis was successfully detected in 69 of the tumor samples.

§ also provided information for the outcome in wild-type populations.

# an increased GCN was defined as an average of three or more locus copies per nucleus, or a locus to centromere ratio of two or more.

M, male; F, female; FISH, fluorescent *in situ* hybridization; CISH, chromogenic *in situ* hybridization; qPCR, quantitative polymerase chain reaction; SISH, silver *in situ* hybridization; NR, not reported; OS, overall survival; PFS, progression-free survival; TTP, time-to-progression.

Eight of the studies employed FISH, one employed CISH and two employed both methods, two employed silver *in situ* hybridization (SISH) and quantitative polymerase chain reaction (qPCR), respectively (Table 1). Gene copy number was scored/assessed according to a different cutoff value, which usually was derived from the receiver operating characteristics (ROC) curve analysis. Twelve studies were retrospective and one was prospective. All eligible studies were small, with sample sizes ranging from 20 to 277 patients (median size = 86 patients, mean size = 92 patients, standard deviation = 67). Overall, the eligible studies reported on 1174 patients, of whom 407 (35%) were characterized as having increased EGFR gene copy number. The frequency increased EGFR gene copy number ranged from 15% to 77%. Eleven of the studies were conducted in European (1058 patients, 338 with increased gene copy number; 32%) whereas two were conducted in East Asian populations (116 patients, 69 with increased gene copy number; 59%). Among all the studies, only three were conducted in wild-type colorectal cancer patients, but two provided data for the outcome in wild-type populations.

### Meta-analysis Database

**Figure 2:**
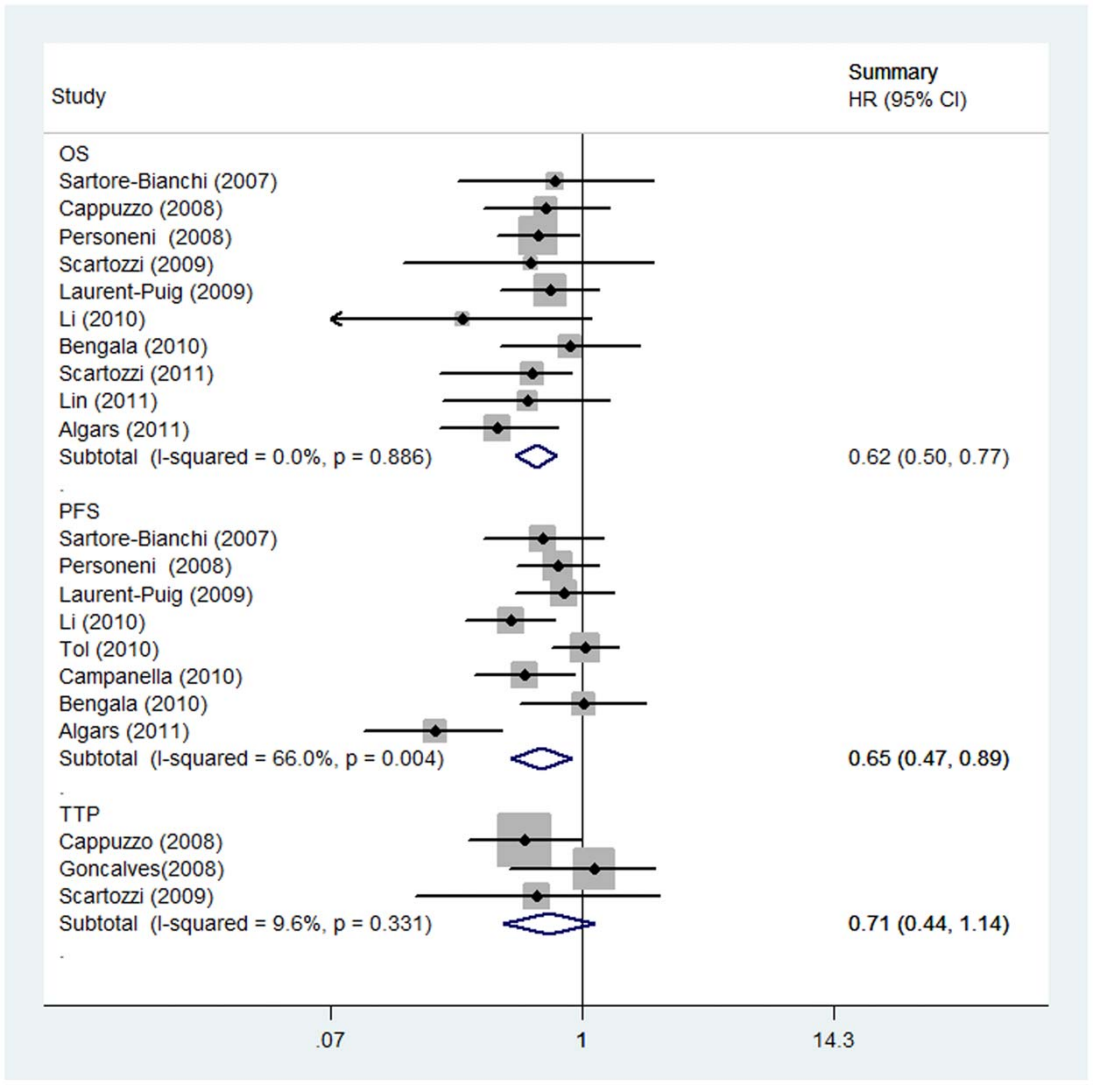
Forest plot for survival stratified by overall survival (OS), progression-free survival (PFS) and time-to-progression (TTP). Hazard ratios (HR) comparing patients with increased versus not increased EGFR gene copy number are presented. Each study is shown by the point estimate of the HR (square proportional to the weight of each study) and 95% confidence interval for the HR (extending lines); summary HR and their 95% confidence intervals by random-effects calculations are shown by diamonds. Value lower than one indicates that patients with increased EGFR gene copy number have improved survival compared to patients without increase in EGFR gene copy number.

Regarding OS, 10 studies involving 776 patients (302 with increased gene copy number, 39%) contributed data for the meta-analysis. There was no between-study heterogeneity (*P* = 0.886; *I^2^* = 0.0%) and increased GCN was significantly associated with improved OS among patients treated with anti-EGFR mAbs (HR = 0.62; 95% CI 0.50–0.77; *P*<0.001) (Figure 2). For PFS, 8 studies involving 893 patients (282 with increased gene copy number; 32%) contributed data for the meta-analysis. Large between-study heterogeneity was observed (*P* = 0.004; *I^2^* = 66.0%) and increased EGFR GCN was significantly associated with improved PFS (HR = 0.65; 95% CI 0.47–0.89; *P* = 0.008). Finally, for TTP, only three studies (149 patients, 66 with increased gene copy number, 44%) provided information to be included in the meta-analysis. There was no between-study heterogeneity (*P* = 0.331; *I^2^* = 9.6%) and we did not find a significant TTP benefit for patients with increased EGFR GCN (HR = 0.71; 95% CI 0.44–1.14; *P* = 0.157).

### Subgroup Analysis

The results of subgroup analysis are presented in Table 2. Increased EGFR GCN was statistically significantly associated with increased OS and PFS in studies of ≥80% populations received second-line or higher but not <80%, and the positive association was also shown in those populations with KRAS mixed status or wild-type, which suggested EGFR GCN might be an independent prognostic factor. Moreover, positive correlations of increased GCN with OS and PFS were shown in the various ethnicities, anti-EGFR mAbs and detection methods, and no significant difference existed between these subgroups.

**Table 2:** Subgroup analyses for overall and progression-free survival for treatment with anti-EGFR drugs, comparing patients with increased versus not increased EGFR copynumber.

Comparison	Overall survival		Progression-free survival	
	Number of studies, heterogenrity (*P_Q_*; *I^2^*)	HR (95% CI); *P* value	Number of studies, heterogenrity (*P_Q_*; *I^2^*)	HR (95% CI); *P* value
All studies	10(0.886; 0)	0.616(0.495–0.766); <0.001	8(0.004; 66)	0.651(0.474–0.894); 0.008
Ethnicity				
White	8(0.885; 0)	0.634(0.504–0.796); <0.001	7(0.008; 65.6)	0.784(0.616–0.907); 0.003
East Asian	2(0.405; 0)	0.458(0.219–0.961); 0.039	1(NA)	0.470(0.292–0.756); 0.002
KRAS status				
wild-type	5(0.504; 0)	0.545(0.388–0.766); <0.001	3(0.002; 83.9)	0.345(0.130–0.919); 0.033
mixed	5(0.461; 0)	0.575(0.428–0.771); <0.001	6(0.001; 75.2)	0.688(0.564–0.840); <0.001
anti-EGFR mAbs				
cetuximab	7(0.945; 0)	0.624(0.485–0.803); <0.001	5(0.074; 53.2)	0.746(0.610–0.916); 0.004
others	3(0.269; 23.8)	0.593(0.384–0.915); 0.018	3(0.006; 80.5)	0.551(0.373–0.814); 0.003
Detection method				
FISH	5(0.703; 0)	0.660(0.488–0.894); 0.007	6(0.096; 46.5)	0.747(0.614–0.910); 0.004
others	5(0.780; 0)	0.572(0.418–0.782); <0.001	2(0.003; 88.7)	0.517(0.339–0.791); 0.002
Line of treatment				
<80% second-line or higher	1(NA)	0.880(0.419–1.847); 0.735	3(0.121; 52.7)	0.874(0.667–1.146); 0.330
≥80% second-line or higher	9(0.908; 0)	0.596(0.474–0.748); <0.001	5(0.019; 66.1)	0.590(0.465–0.748); <0.001

Subgroup analyses was performed when at least two studies were in each subgroup.

Subgroup analysis was not performed for TTP as only three studies provided information for this outcome.

FISH, fluorescent *in situ* hybridization; NA, not applicable; CI, confidence interval; HR, hazard ratio.

### Test of Heterogeneity

There was extreme heterogeneity among the 8 studies including PFS (*I^2^* = 66, *P* = 0.004). Therefore, we performed a meta-regression analysis to evaluate the source of heterogeneity by ethnicity, KRAS status, anti-EGFR mAbs, detection method and line of treatment. However, when we categorized the heterogeneity by these factors, none of these significantly contributed to the observed heterogeneity.

### Sensitivity Analysis

Sensitivity analysis was performed both by sequential remove of individual studies and cumulative statistics for all comparisons of all subjects and subgroups. It was shown that with the passage of time and increasing the sample size, the results of OS and PFS become more stable. The pooled HRs were not inﬂuenced by any individual study.

### Publication Bias

Funnel plots and Egger’s test were performed to assess publication bias. The data suggested that there was no evidence of publication bias for the study’s primary outcome, OS (Begg’s test *P* = 0.53; Egger’s test *P* = 0.46), PFS (Begg’s test *P* = 0.32; Egger’s test *P* = 0.13), TTP (Begg’s test *P* = 0.60; Egger’s test *P* = 0.85).

## Discussion

In the present study, we collected all available studies and carried out a meta analysis to examine the association of variations of EGFR GCN with prognosis of advanced CRC patients. Ten studies involving 776 patients on OS, eight studies involving 893 patients on PFS and three studies on TTP were critically reviewed. We subgrouped the articles into five groups (ethnicity, KRAS status, anti-EGFR mAbs, detection method and line of treatment). Meta-analysis showed increased EGFR GCN was significantly associated with improved OS and PFS but not TTP. The median OS of patients harboring increased GCN showed 1.61-fold increase, the median PFS showed 1.54-fold increase. A meta-analysis of these studies confirms that increased EGFR GCN is indeed associated with a moderate OS and PFS benefit, from anti-EGFR treatment for metastatic CRC patients. Similarly, EGFR gene copy number has also been evaluated as a potential predictor of response of tyrosine kinase inhibitors (TKIs) in non-small-cell lung cancer patients, and a meta analysis has demonstrated an association between increased EGFR copy number, and improved survival outcomes [31]. Recently, Yang *et al.* performed a meta analysis to differentiate the objective response rate (ORR) between patients with increased EGFR GCN and those with no increased EGFR GCN [32]. They suggested a general trend towards higher ORR in patients with increased EGFR GCN. However, for important prognostic factors as PFS and OS, as the data was relatively incomplete, they only descriptively reviewed published papers and did not perform quantitative synthesis of the studies. In this study, several excellent HR extraction methods were used to calculate the pooled HR quantitatively. The result showed increased EGFR GCN association with improved survival outcomes among anti-EGFR-treated patients. These results imply the EGFR GCN might be not only an effective predictive but also a valuable prognostic marker.

The anti–EGFR monoclonal antibody is effective in prolonging survival in patients with metastatic colorectal cancer after failure of conventional chemotherapy [33], [34]. In our stratified analysis, the increased EGFR GCN was significantly associated with improved OS and PFS in those populations that received second-line or higher but not first-line, which coincided with the strategies in clinical practice of chemotherapy. From a clinical point of view, not only in the US and Europe but also in China, anti-EGFR mAbs were usually used in wild-type KRAS mCRC patients. So, assessing the role of EGFR GCN in patients with wild-type KRAS may be more meaningful. In this study, we found that the prognostic value of EGFR GCN on survival appears to not be related to KRAS status, which suggested EGFR GCN might be an independent prognostic biomarker. The significant association between survival with EGFR GCN, revealed tumor growth is probably mainly driven by the EGFR pathway and this biological characteristic is evoked by an increase in EGFR copy number.

EGFR is a transmembrane tyrosine kinase receptor that, on ligand binding, triggers two main signaling pathways, the RAS-RAF-MAPK axis, which is mainly involved in cell proliferation, and the PI3K-PTEN-AKT pathway, which is mainly involved in cell survival and motility [35]. The anti-EGFR mAbs have been proven to be effective in metastatic colorectal cancer. The molecular mechanisms underlying the clinical response to this drug remain unknown. Genetic alterations in EGFR-related signaling pathways may have an effect on response to this targeted therapy, which may be due to the constitutive activation of the downstream genes of the EGFR signaling pathway such as KRAS, BRAF, or PIK3C2A, or to the loss of a tumor suppressor gene such as PTEN. Until now, the most acceptable marker, as a predictive and prognostic factor, was the status of KRAS. However, KRAS was not the only predictor of the cetuximab response. The present study was aimed at assessing the prognostic role of EGFR GCN, in terms of clinical outcome, in patients treated with anti-EGFR mAbs. EGFR GCN detection also appears to be relevant to positively identify responders. Variations of GCN, reflect the many different routes taken by individual tumors to disrupt/escape mechanisms governing normal cellular behavior. In most solid tumors, including CRC, the best characterized mechanisms underlying increased EGFR GCN are gene amplification and chromosome 7 polysomy [9], [10], [12], [36].

Current obstacles for a future clinical application of EGFR GCN are mainly concentrated on the following two aspects: detection methods and difficult technical reproducibility. FISH technique has been used in most previous studies, but the FISH results are challenging to interpret and the lack of standardization of analytical methods and scoring systems may partly explain why the EGFR GCN evaluation has not been incorporated into clinical practice yet [37]. When looking at the different cutoff values in the literature, we found reproducibility remains a large obstacle for its practical usefulness and an international consensus on the definition of cutoff points is needed. Sartore-Bianchi *et al.* also found that molecule diagnosis of EGFR GCN by FISH among five highly experienced pathology centers varied largely, a detailed scoring system and comprehensive training programmes are necessary [38]. Although different cutoff points have been applied, 95% CIs around sensitivity and specificity yielded by each cutoff point were similar, thus indicating that results from these studies are consistent. In the present study, we analyzed the influence of GCN detection method on survival and did not found any discrepancies.

There are several limitations kept in consideration in this meta-analysis. First, most of the studies were not conclusive because they evaluated limited patient series that were nonhomogeneously treated. Second, relatively small sample sizes included in East Asians may also inﬂuence the results, and further studies are necessary to detect the potential role of GCN. Third, primarily the unavailability of individual patient data that would allow correction for potential confounding factors such as age, gender, or additional genetic aberrations. Finally, different detection methods used in the studies included in the analysis may have different quality control issues.

In conclusion, our meta-analysis provides evidence that EGFR gene copy number is a prognostic marker for survival among patients receiving anti-EGFR mAbs for advanced colorectal cancer. Furthermore, according to our results, the prognostic ability of EGFR gene copy number appears to be significantly stronger among those populations that received second-line or higher treatment.
